# Barriers and facilitators to the uptake of computerized clinical decision support systems in specialty hospitals: protocol for a qualitative cross-sectional study

**DOI:** 10.1186/s13012-014-0105-0

**Published:** 2014-08-28

**Authors:** Lorenzo Moja, Elisa Giulia Liberati, Laura Galuppo, Mara Gorli, Marco Maraldi, Oriana Nanni, Giulio Rigon, Pietro Ruggieri, Francesca Ruggiero, Giuseppe Scaratti, Alberto Vaona, Koren Hyogene Kwag

**Affiliations:** Dipartimento di Scienze Biomediche per la Salute, Università degli Studi di Milano, Via Pascal 36, Milan, 20133 Italy; Unità di Epidemiologia Clinica, IRCCS Istituto Ortopedico Galeazzi, Via R. Galeazzi 4, Milan, 20161 Italy; Dipartimento di Psicologia, Università Cattolica del Sacro Cuore di Milano, L.go Gemelli 1, Milan, 20123 Italy; Clinica Ortopedica e Traumatologica II, IRCCS Istituto Ortopedico Rizzoli, via G.C.Pupilli 1, Bologna, 40136 Italy; IRCCS Istituto Scientifico Romagnolo per lo Studio e la Cura dei Tumori, Via Piero Maroncelli 40, Meldola, 47014 FC Italy; Azienda ULSS 20, P.le Lambranzi 1, Verona, 37034 Italy

**Keywords:** Computerized clinical decision support systems, Evidence-based medicine, Perceptions, Qualitative

## Abstract

**Background:**

Computerized clinical decision support systems (CDSSs) have been shown to improve the efficiency and quality of patient care by connecting healthcare professionals with high quality, evidence-based information at the point-of-care. The mere provision of CDSSs, however, does not guarantee their uptake. Rather, individual and institutional perceptions can foster or inhibit the integration of CDSSs into routine clinical workflow. Current studies exploring health professionals’ perceptions of CDSSs focus primarily on technical and usability issues, overlooking the social or cultural variables as well as broader administrative or organizational roles that may influence CDSS adoption. Moreover, there is a lack of data on the evolution of perceived barriers or facilitators to CDSS uptake across different stages of implementation.

**Methods:**

We will conduct a qualitative, cross-sectional study in three Italian specialty hospitals involving frontline physicians, nurses, information technology staff, and members of the hospital board of directors. We will use semi-structured interviews following the Grounded Theory framework, progressively recruiting participants until no new information is gained from the interviews.

**Discussion:**

CDSSs are likely to become an integral and diffuse part of clinical practice. Various factors must be considered when planning their introduction in healthcare settings. The findings of this study will guide the development of strategies to facilitate the successful integration of CDSSs into the regular clinical workflow. The evaluation of diverse health professionals across multiple hospital settings in different stages of CDSS uptake will better capture the complexity of roles and contextual factors affecting CDSS uptake.

## Background

Evidence-Based Medicine (EBM), the integration of individual clinical expertise with evidence from scientific research [1], is widely regarded as a key driver of continuous improvement in healthcare services. Studies, however, consistently report the limited success of healthcare interventions in transferring evidence-based knowledge into clinical practice [2]. In the last years, extensive investments have been made to develop technologies that will better connect health professionals with high-quality research. One such technology that is gaining momentum is the computerized clinical decision support system (CDSS). A CDSS can be defined as an information system aimed to support clinical decision-making, which links patient-specific information in electronic health records (EHRs) with evidence-based knowledge to generate case-specific guidance messages through a rule- or algorithm-based software [3].

Current studies shows that CDSSs have the potential to improve the efficiency and quality of patient care by increasing, for instance, the safety of medication prescribing, use of preventative care in hospitalized patients, access to accurate medical records, patient-physician communication, and adherence to guideline-based care [4]–[6]. The effectiveness of CDSSs on patient outcomes and healthcare utilization, including morbidity, mortality, and economic outcomes is less clear [3]. New generations of CDSSs can replicate the natural flow of thought for clinical decision-making at the point-of-care where online evidence is translated into patient-specific recommendations [7]. CDSSs offer a solution to many frequently cited barriers in evidence-based practice such as time constraints and difficulties in navigating complex information systems and interpreting results [8]. Information that is accessible at the point-of-care is seen by clinicians as a key factor in the successful incorporation of evidence in routine care [9]. Despite consistent findings demonstrating the potential of CDSSs to improve patient outcomes and health professional behavior, the mere provision of the technology does not guarantee its uptake. In fact, even if a CDSS is readily available within a hospital, clinicians often fail to adopt its recommendations, ignoring up to 96% of its alerts [10]. Our study aims to detect the barriers and facilitators to CDSS uptake as perceived by diverse health professionals in specialty hospitals at different stages of the technology’s implementation.

With some exceptions (e.g., Moxey et al. [10]), most studies examining health professionals’ perception of CDSSs report usability, either technical or pragmatic (*e.g*., lack of access to personal computers or hardware devices), as a key facilitator or barrier to the acceptance and use of CDSS [9],[11],[12]. Although these issues may be important to regulating CDSS uptake, less overt, but overarching reasons such as the local practice culture or an institution’s openness to EBM can contribute to the technology’s implementation. CDSSs materialize the evidence-based paradigm at the point-of-care, encouraging health professionals to be accountable for the use of evidence in their practice. Berg [13] suggests that the introduction of CDSSs is a part of a much broader historical, institutional, and political ambition to make medical decisions more rational and less discretional. The EBM paradigm is often rejected because it challenges well-established hierarchies and systems of power based on seniority [14]. For example, Bhandari and colleagues [15] found that surgical residents did not implement evidence-based practices for fear of repercussions from staff members. Christakis and Rivara [16] found that physicians disliked guidelines, regarding them as capable of devaluing physicians’ ability to determine what is best for their patient. These studies suggest that the fear of compromising clinical expertise and medical judgment, challenging previous preferences and practices, and disrupting the local clinical workflow may cause health professionals and institutions to resist EBM [14],[17]. Clinicians seem to be more willing to rely on their communities of practice rather than on guidelines to form their medical decisions: consolidated ‘mindlines’, tacit guidelines that are collectively reinforced and internalized by a network of colleagues or other trusted sources, might be more powerful in directing the course of medical practice compared to evidence [18].

The complexity of introducing new technologies into healthcare settings may play an additional role in CDSS uptake. The integration of a formal tool into an organized context requires negotiating with all involved parties and addressing the particularities of specific workplaces. When a technology is implemented in a workplace, the two shape and transform each other through an iterative process [13],[19]. Berg’s studies on the introduction of technologies in healthcare settings, including decision support systems [13], clinical protocols [13], and guidelines [20], suggest the need to go beyond approaches centered on the ‘fit’ between technology systems and healthcare organizations. Rather, the acceptance of technologies by these organizations is a social process based on inter-professional negotiations [21],[22] in which health professionals’ attitudes are negotiated and disciplined to the technology, and vice versa [13],[19].

The introduction of information technologies in clinical settings, even when highly promising, is far from straightforward. Social, cultural and contextual factors, together with clinicians’ perceptions and understandings of information technologies, can influence the outcome of CDSS introduction in healthcare settings. Therefore, CDSS implementation needs to be analyzed with a socio-technical approach [23] that is sensitive to the multiple, and perhaps contradictory, ways in which CDSSs are interpreted, used, and shaped by diverse healthcare professionals within their working contexts.

### The research gap

After reviewing the literature on the perceived facilitators and barriers to CDSS implementation of health professionals, we identified three main limitations to previous studies. First, the studies largely focused on technical and usability issues, while they tended to overlook the social, cultural and contextual factors potentially influencing their implementation [9],[11],[12],[24],[25]. Moxey and colleagues [10] suggested that the variability in CDSS uptake may be attributable to the technical aspects of the technology itself. Second, most studies evaluated the perceptions of frontline clinicians, but did not address the perceptions of different organizational roles (*e.g*., hospital administrators, chiefs, or non-physician staff) that are key to establishing the overall mission and vision of the healthcare institution in addition to shaping the expected behavior and standards of its personnel. Organizational leadership that is supportive of technological innovation, for example, may encourage and reward the use of CDSSs to improve patient care. Third, the studies often addressed contexts in which CDSSs had already been introduced; these studies did not account for the perceived facilitators and barriers existing prior to CDSS introduction, or for the evolution of perceptions throughout the technology’s various stages of uptake. These limitations are especially relevant in countries such as Italy where the majority of healthcare contexts have not yet adopted CDSSs.

Our study seeks to identify potential barriers and facilitators to the adoption of EBM-focused CDSSs linked to EHRs in specialty hospitals. We address the limitations of previous studies by considering: a) multiple health professionals, including physicians, nurses, and hospital managers, and b) hospitals with different levels of CDSS infrastructure and use as well as experience in implementing EBM.

## Methods

We will conduct a qualitative, cross-sectional study based on semi-structured interviews to examine individual and contextual barriers and facilitators to CDSS uptake. The interview will be designed and analyzed accordingly to the constructivist Grounded Theory (GT) approach, the inductive development of theory from data [26]. Instead of initiating the study with a hypothesis or research question, the GT method begins with empirical observations on the field of interest [26]. Data on the main features, conditions, outcomes, and contextual factors of the object of study is used to ground and systematically generate a theory surrounding the social or socio-psychological process [26],[27]. By adopting this approach, we aim to capture the complex and multi-dimensional processes and relationships involved in the use and adoption of CDSSs across different stages of implementation.

One of the main elements of the GT method is the collection of data before its analysis. The former is driven by theoretical sampling, the sequential selection of individuals in a study sample according to the state of theory generation [27]. Following the criteria of theoretical saturation [28], we will progressively recruit participants until no new information is raised from the interviews.

### Setting and participants

We will adopt a purposive sampling strategy and select the first participants using a maximum variability logic [29]. This strategy will allow us to explore contexts with different levels of familiarity with CDSSs as well as participants with diverse organizational roles.

To determine the impact of particular clinical setting characteristics on CDSS uptake, we selected three specialty research hospitals located in northern Italy based on the following criteria: the hospital reported the use of evidence during practice, an EHR, and a level of CDSS implementation and familiarity. Specifically, setting ‘A’ is an oncology hospital that abandoned paper-based clinical documents and fully adopted an EHR beginning in 2008. The hospital’s EHR is linked to a variety of CDSSs, including evidence-based messages on treatment and diagnosis using care management algorithms. All participants from setting ‘A’ will be considered current users of CDSSs and compliant with EBM. Setting ‘B’ is an orthopedic research hospital that has been using an EHR since 2011, but does not have a CDSS. In fact, this hospital’s EHR is not sufficiently developed to link to a CDSS or to be utilized by health professionals who have adopted standard international codes, a prerequisite to the activation of CDSS guide messages. Health professionals in setting ‘B’ may or may not be compliant with EBM depending on their own capacity and willingness. Setting ‘C’ is an orthopedic research hospital that does not have an EHR or a CDSS. This setting will be considered an environment reluctant to innovation and the use of evidence in practice.

In order to determine the impact of particular professional or organizational roles and characteristics on CDSS perception, we will interview frontline physicians, nurses, information technology staff, and members of the hospital board of directors. We will use purposive sampling to capture the perspectives of a diverse and representative sample of professionals.

The demographics or seniority of participants will not be considered in the selection criteria; nonetheless, this information will be collected during the interview. Table 1 shows the purposive sample we will use to collect the data. Consistent with the theoretical sampling strategy, we will add to our sample throughout the data collection process based on the provisional results from the analyses. For example, we will increase the number of participants in one cluster of stakeholders, or include health professionals that were not expected to participate in the sample if their positions and experiences require further consideration or elaboration.

**Table 1:** **First stage of purposive sampling**

Settings			Participants		
	Technological familiarity and readiness		Decision-makers	Frontline practitioners	
	CDSS	EHR	IT staff and members of the board of directors	Physicians	Nurses
*A*	Yes	Yes	>1	>3	>3
*B*	No	Yes	>1	>3	>3
*C*	No	No	>1	>3	>3

### Data collection

Data will be collected through interviews in hospital consultation rooms. The interview will address the following topics: a) participants’ beliefs and experiences with information technology, in general (*e.g*., the use of personal computers, tablets, and smartphones to obtain information relevant to their clinical practice); b) beliefs and experiences with CDSSs, specifically; c) willingness to adopt EBM and clinical guidelines in their routine practice; and d) perceptions regarding the potential of CDSSs to integrate evidence and guidelines in clinical practice. We will ask participants to discuss both their own experiences and those of their colleagues in their workplace.

We will follow the GT principle of progressively refining the interview framework according to participants’ answers around the object of interest. In other words, interviews will not follow a prescribed structure; rather, questions will be developed continuously for each interview. Participants will be contacted by the three Unit Coordinators (LM, MM, ON) and invited to participate in the study. Each interview will be conducted by two individuals: a trained investigator who will conduct the interview, and a medical doctor with competence in the participating hospital’s field of specialty who will support the investigator in clinical topics. The expected duration of the interview is 30 to 90 minutes. The interviews will be taped and transcribed verbatim.

### Data analysis

Investigators will analyze all interview transcripts according to the procedure outlined in GT content analysis, which involves three sequential phases of coding [27],[28]. In the first step, open coding, investigators will identify and label preliminary concepts found in the data (*e.g*., `CDSS is less reliable than colleagues’). Investigators will analyze the interview transcripts line-by-line to detect ‘in vivo’ codes that directly use the participant’s wording. In axial coding, the second analytical step, investigators will reassemble particular sets of data based on central concepts that emerge from the ongoing analysis; in other words, codes will be progressively aggregated into broader categories (*e.g*., ‘resilience of paper-based culture’ or ‘power and hierarchy issues’). This step involves the recurrent identification and comparison of themes both within and across sub-categories and broader categories. In selective coding, the final step of data analysis, investigators will further define, develop and refine discrete concepts and categories. The core categories, pivotal concepts encapsulating the whole phenomenon under investigation will be selected and systematically related to the other categories. The combined categories and their interrelationships will ultimately form a larger storyline surrounding the process of CDSS uptake [27],[28].

The coding process will be conducted by three investigators (EGL, LM, MG). The NVivo software (version 10) [30] will be used to support the analysis.

### Ethics and funding

The study was approved by the Research Ethics Committees of IRCCS Istituto Ortopedico Rizzoli (approved January 31, 2014; file number 0003938/2014), IRCCS Ospedale San Raffaele (approved March 6, 2014), and IRCCS Istituto per la Cura dei Tumori della Romagna (approved February 21, 2014; file number 1155/2014). This work is supported by the Italian Ministry of Health (GR-2009-1606736) and by Regione Lombardia (D.R.G. IX/4340 26/10/2012). Funding sources had no role in the writing of this manuscript or the decision to submit it for publication.

Before beginning the interview, participants will be given an informed consent form as well as information outlining the purpose of the study and participant rights. Participants will be notified that their involvement is voluntary and can be withdrawn at any time, and that confidentiality is protected through the anonymization of all collected data.

## Discussion

CDSSs are likely to become an integral part of clinical practice in the endeavor for continual improvement in patient care and safety. Well-established clinical workflows and EHRs are important requisites to the successful introduction and uptake of CDSSs in clinical settings. Users should also be provided with sufficient training, education and support. In addition to developing technical suggestions on CDSS design and implementation (*e.g*., keep alerts simple, straightforward, and specialized to the area of use) [31], the understanding of perceived barriers and facilitators to CDSSs is important to maximize the technology’s usage and potential to impact patient outcomes.

We propose a dynamic and integrative model for studying CDSSs that considers a wide range of contexts, from healthcare organizations that are unfamiliar with the technology to those in mature stages of its implementation. The inclusion of diverse stakeholders beyond frontline clinicians will capture the complexity of the roles and responsibilities influencing CDSS uptake. This may further foster trust, transparency and cooperativeness in healthcare settings, contributing to the increased acceptance and use of CDSSs. The results of this study will guide the development of strategies and recommendations for the successful introduction and integration of CDSSs into healthcare organizations.

A limitation of the study is the collection of data through interviews rather than real-life settings in which health professionals directly engage with CDSSs. The observation of health professionals in their practice would require longer and a more demanding access to the specialty hospital that has adopted CDSSs. Moreover, we believe that observing CDSS use is an insufficient trigger for a detailed investigation of the understandings, beliefs and attitudes on the technology and evidence-based practice. However, to address the limitations of our methodological choice, we created a three-minute video that will be shown to health professionals during the interviews. The video introduces the main functions and features of the CDSS that distinguishes it from other similar technologies. This video is intended to provide basic information to participants who are unfamiliar with the tool, and encourage concrete and practical reflections on the issues related to CDSS use in routine clinical activities.

We anticipate several challenges in the running of this trial. Because the study involves multiple sites, an effective communication strategy will be necessary to promote optimal communication between the investigators and researchers. We will schedule regular teleconferences and face-to-face meetings to share updates on the progress of the study at each site. We will use the NVivo software to share data and information for data analysis. We will further develop an instructional manual with clear procedures for the sharing and handling of data in order to ensure its security, integrity and quality.

Additionally, there may be difficulties in coordinating interview schedules for busy health professionals while maintaining the consistency and accuracy of the data collection process. We anticipate that the structure, setting or nature of an interview itself may influence participants’ responses. Investigators will attempt to make the interview resemble a natural conversation as much as possible so that participants can freely discuss their experiences and beliefs. We will also verbally inform participants that the purpose of the study is not to evaluate the staff, but to explore their beliefs and experiences with CDSSs and evidence-based practice.

Future studies can focus longitudinally on CDSS introduction and uptake in order to determine the long-term effects of the intervention.

## Abbreviations

CDSS: Clinical decision support systems
EBM: Evidence-based medicine
EHR: Electronic health record
GT: Grounded theory
